# Supplementary material for: NMR study of the interaction between MinC and FtsZ and modeling of the FtsZ:MinC complex
Source: J Biol Chem. 2025 Jan 9;301(3):108169. doi: 10.1016/j.jbc.2025.108169 (PMC11938149; doi:10.1016/j.jbc.2025.108169)
Supplement: Tables S1-S5 and Figures S1-S4 [file mmc1.docx]

**SUPPORTING INFORMATION**

**NMR study of the interaction between MinC and FtsZ and modeling of the FtsZ:MinC complex.**

Luciana E. S. F. Machado^a#^, Patricia Castellen^a,b#^, Valdir Blasios^a^, Helder V. Ribeiro-Filho^b^, Alexandre W. Bisson-Filho^a^, Jhonatan S. Benites Pariente^a^, Maria L. C. Nogueira^b^, Mauricio Sforça^b^, Rodrigo V. Honorato^b^, Paulo S. Lopes-de-Oliveira^b^, Roberto K. Salinas^a^, Ana C. Zeri^b^, Frederico J. Gueiros-Filho^a^.

This PDF file includes:

Supplemental Tables S1-S5

Supplemental Figures S1-S4

Supplemental References

**Table S1.** *B. subtilis* strains.

| **Strain** | **Genotype** | **Source** | **Figure** |
| --- | --- | --- | --- |
| **PY79** | *prototroph* | (Youngman et al., 1983) | WT parent strain |
| **FG1454** | *minCD::spc thr::P_spac_-gfp-minC_minD (erm)* | this work | S2A, S2B |
| **VB28** | *minCD::spc thr::P_spac_-gfp-minC^K12A^_minD (erm)* | this work | S2A, S2B |
| **VB29** | *minCD::spc thr::P_spac_-gfp-minC^K15A^_minD (erm)* | this work | S2A, S2B |
| **VB45** | *minCD::spc thr::P_spac_-gfp-minC^Y8A^_minD (erm)* | this work | S2A, S2B |
| **VB47** | *minCD::spc thr::P_spac_-gfp-minC^H55A^ _minD (erm)* | this work | S2A, S2B |
| **VB49** | *minCD::spc thr::P_spac_-gfp-minC^H84A^_minD (erm)* | this work | S2A, S2B |

**Table S2.** Plasmids.

| **Plasmid** | **Genotype** | **Source** | **Figure** |
| --- | --- | --- | --- |
| **pAT6** | *minC^1-102^ in pET28a (kan)* | (Castellen et al., 2015) | 1, S1 |
| **pAB20** | *ftsZ^1-382^ in pET28a* | (Blasios et al., 2013) | 4 |
| **pAB30** | *minC^1-224^ in pET24b* | (Blasios et al., 2013) | 4 |
| **pBLA1** | *minC^K12A^ in pET24b (kan)* | this work | 4 |
| **pBLA2** | *minC^K15A^ in pET24b (kan)* | this work | 4 |
| **pBLA6** | *minC^Y8A^ in pET24b (kan)* | this work | 4 |
| **pBLA7** | *minC^H55A^ in pET24b (kan)* | this work | 4 |
| **pBLA8** | *minC^H84A^ in pET24b (kan)* | this work | 4 |
| **pBLA10** | *thr::P_spac_-gfp-minC^K12A^_minD (erm)* | this work | S2A, S2B |
| **pBLA11** | *thr::P_spac_-gfp-minC^K15A^_minD (erm)* | this work | S2A, S2B |
| **pBLA14** | *thr::P_spac_-gfp-minC^Y8A^_minD (erm)* | this work | S2A, S2B |
| **pBLA15** | *thr::P_spac_-gfp-minC^H55A^ _minD (erm)* | this work | S2A, S2B |
| **pBLA16** | *thr::P_spac_-gfp-minC^H84A^ _minD (erm)* | this work | S2A, S2B |
| **pLM1** | *minC^1-102^ in pRP1B (kan)* | this work | 2, 3 |
| **pLM2** | *minC^1-102 K12A^ in pRP1B (kan)* | this work | 2, 3 |
| **pLM3** | *minC^1-102 H55A^ in pRP1B (kan)* | this work | 2, 3 |
| **pLM4** | *FtsZ^1-315, A182E^ in pRP1B (kan)* | this work | 2, 3 |

***Table S3****. Frequency of hydrogen bond formation during MD simulation.*

| **MinC residues** | **FtsZ residues** | **HB frequency (%)** |
| --- | --- | --- |
| LYS5-Main | ASN208-Side | 16.83% |
| LYS15-Side | ASP289-Side | 3.87% |
| LYS2-Side | ASP289-Side | 15.21% |
| TYR8-Side | ASP213-Side | 29.18% |
| LYS4-Side | LEU209-Main | 12.47% |
| LYS5-Side | GLY205-Main | 7.73% |
| SER2-Main | GLY37-Main | 17.21% |
| LYS12-Side | ASP213-Side | 26.31% |
| GLU42-Side | GLN288-Side | 8.73% |
| GLU42-Main | GLN288-Side | 3.99% |
| THR14-Side | GLN288-Main | 4.49% |
| LYS4-Main | ILE201-Main | 1.75% |
| LEU31-Main | GLN276-Side | 1.25% |

***Table S4****. Features of complex´s interfaces estimated using COCOMAPS (https://www.molnac.unisa.it/BioTools/cocomaps/).*

^1^ *Note that our complex contains only MinC^N^ and FtsZ without its unstructured tail, whereas AlphaFold’s used the full-length proteins*.

| **Parameter** | **NMR/Docking** | **AlphaFold** |
| --- | --- | --- |
| Buried area upon complex formation (Å2) | 2353,6 | 2267,7 |
| Buried area upon complex formation (%) ^1^ | 11,41 | 5,77 |
| Interface area (Å2) | 1176,8 | 1133,85 |
| Interface area MOL1 (%) (MinC) ^1^ | 15,15 | 7,49 |
| Interface area MOL2 (%) (FtsZ) ^1^ | 9,15 | 4,69 |
| POLAR Buried area upon complex formation (Å2) | 1447,7 | 1536,2 |
| POLAR Interface (%) | 61,51 | 67,74 |
| POLAR Interface area (Å2) | 723,85 | 768,1 |
| NON POLAR Buried area upon complex formation (Å2) | 905,8 | 731,5 |
| NON POLAR Interface (%) | 38,49 | 32,26 |
| NON POLAR Interface area (Å2) | 452,9 | 365,75 |
| Residues at the interface_TOT (n) | 61 | 50 |
| Residues at the interface_Mol1 (MinC) | 25 | 22 |
| Residues at the interface_Mol2 (FtsZ) | 36 | 28 |
| Number of interacting residues Molecule1 (MinC) | 46 | 44 |
| Number of interacting residues Molecule2 (FtsZ) | 74 | 71 |
| Number of hydrophilic-hydrophobic interaction | 185 | 160 |
| Number of hydrophilic-hydrophilic interaction | 98 | 110 |
| Number of hydrophobic-hydrophobic interaction | 84 | 79 |

***Table S5****. Comparison between E. coli MinC^N^ mutant data and residue presence in interface of predicted complexes.*

^1^ *Residue correspondence as in Park et al. 2018*.

^2^ *Residues which exhibited the same phenotype when mutated in B. subtilis are marked with asterisks.*

^3^ *Contacts predicted by COCMAPS, using a distance threshold of 5Å.*

| **residue**  ***E. coli*** | **residue**  ***B. subtilis***^1,2^ | **Phenotype** | **In our interface?^3^** | **In AF interface? ^3^** |
| --- | --- | --- | --- | --- |
| E7K/E7A | T10 | normal | yes | yes |
| K9A | K12A* | defective | yes | yes |
| G10D | G13 | defective | yes | yes |
| S11D | T14 | normal | no | yes |
| S12D | K15A* | defective | yes | yes |
| S16D | T19 | defective | no | no |
| V18D | H21 | defective | no | no |
| K35E | M38 | defective | yes | no |
| Q38A | I41 | normal | no | yes |
| A39D | E42 | defective | yes | no |
| F42E | T45 | defective | yes | no |


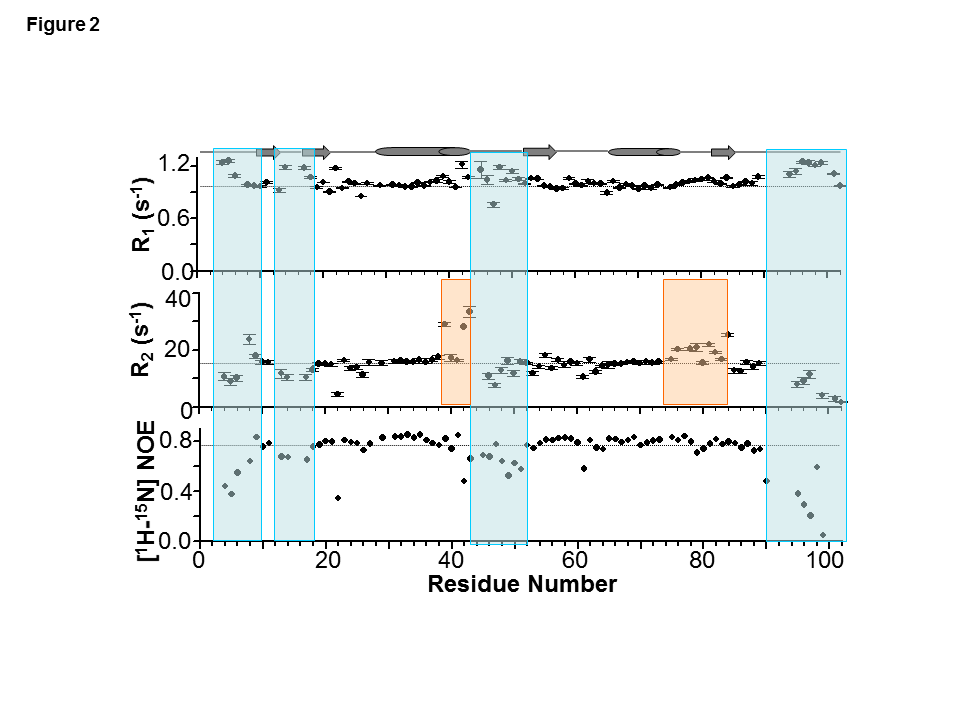


***Supplemental Figure 1****: ^15^N dynamics of MinC^N^. ^15^N R_1_ and R_2_ relaxation rate and hetNOE (^15^N 80 MHz Larmor frequency). Annotated secondary structure is shown. The dynamic loops are highlighted in blue. Regions of potential conformational exchange are highlighted in orange.*


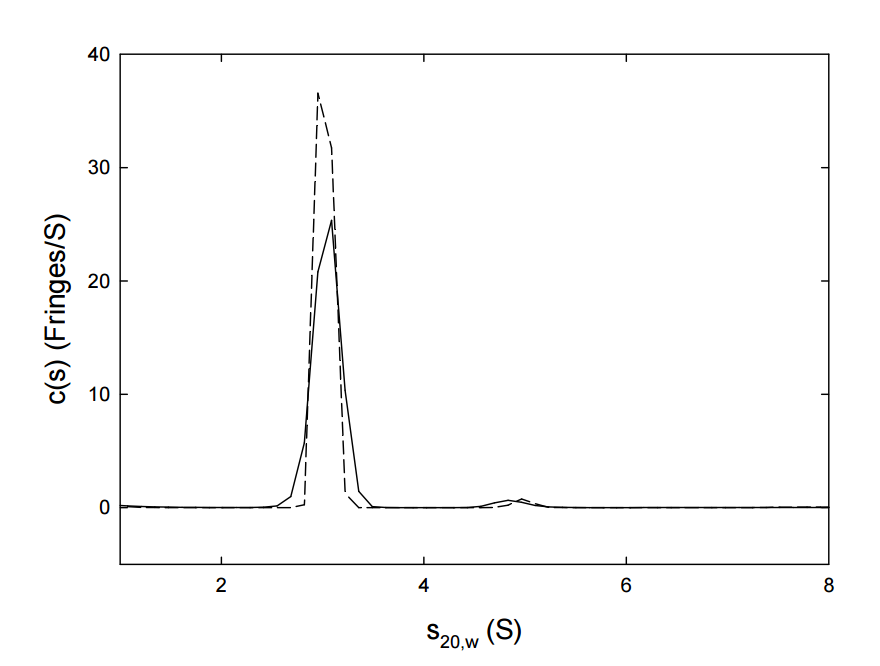


***Supplemental Figure 2:*** Sedimentation coefficient distribution c(s) of 100 μM FtsZ^1-315, A182E^ in the presence of 1.5 mM GDP (dashed line) or 1.5 mM GTP (solid line). More than 97% of the protein sedimented as a single peak with a sedimentation coefficient s_20,w_ value 3.0 S, which is identical to that of the FtsZ^1-315^ monomer.


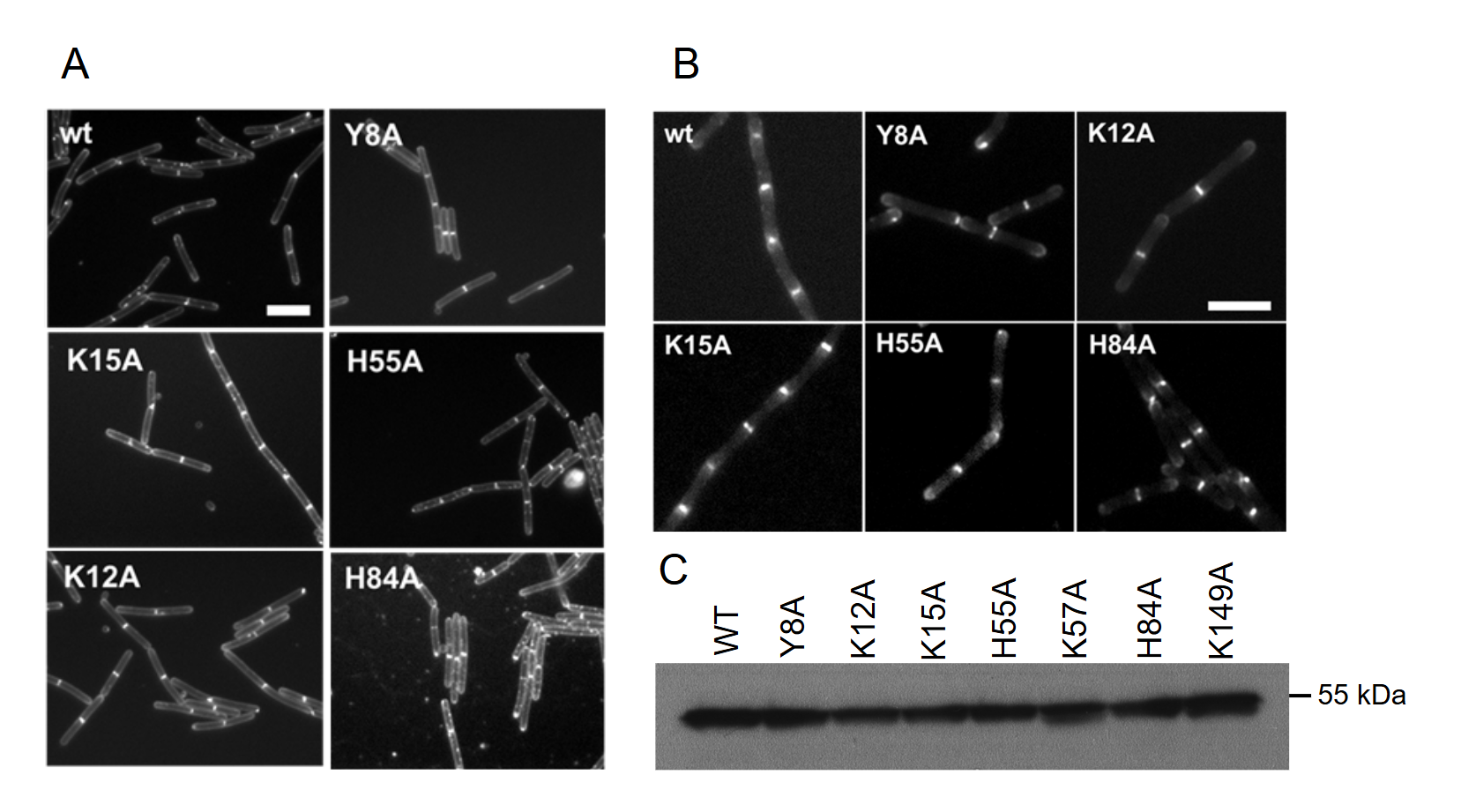


**Supplemental Figure 3***: (A) Cell division phenotype of GFP-MinC^N^ mutants. All strains contain IPTG inducible alleles (Pspac) of GFP-MinC and were grown on LB pads with 0.2 mM of IPTG at 37^o^C for 3 hours. Cells were stained with FM 5-95. Scale bar 5 μm. (B) Subcellular localization of GFP-MinC^N^ mutants. Cells were induced with 0.2 mM IPTG, grown to exponential phase and immobilized on 1% agarose. Scale bar = 5 µm. (C) Western blot of whole cell extracts from each mutant revealed with anti-GFP antiserum. Predicted molecular weight of the GFP-MinC fusions is 52 kDa. Blot contains 2 mutants (K57A and K149A) that are not relevant for this work and will be described elsewhere.*


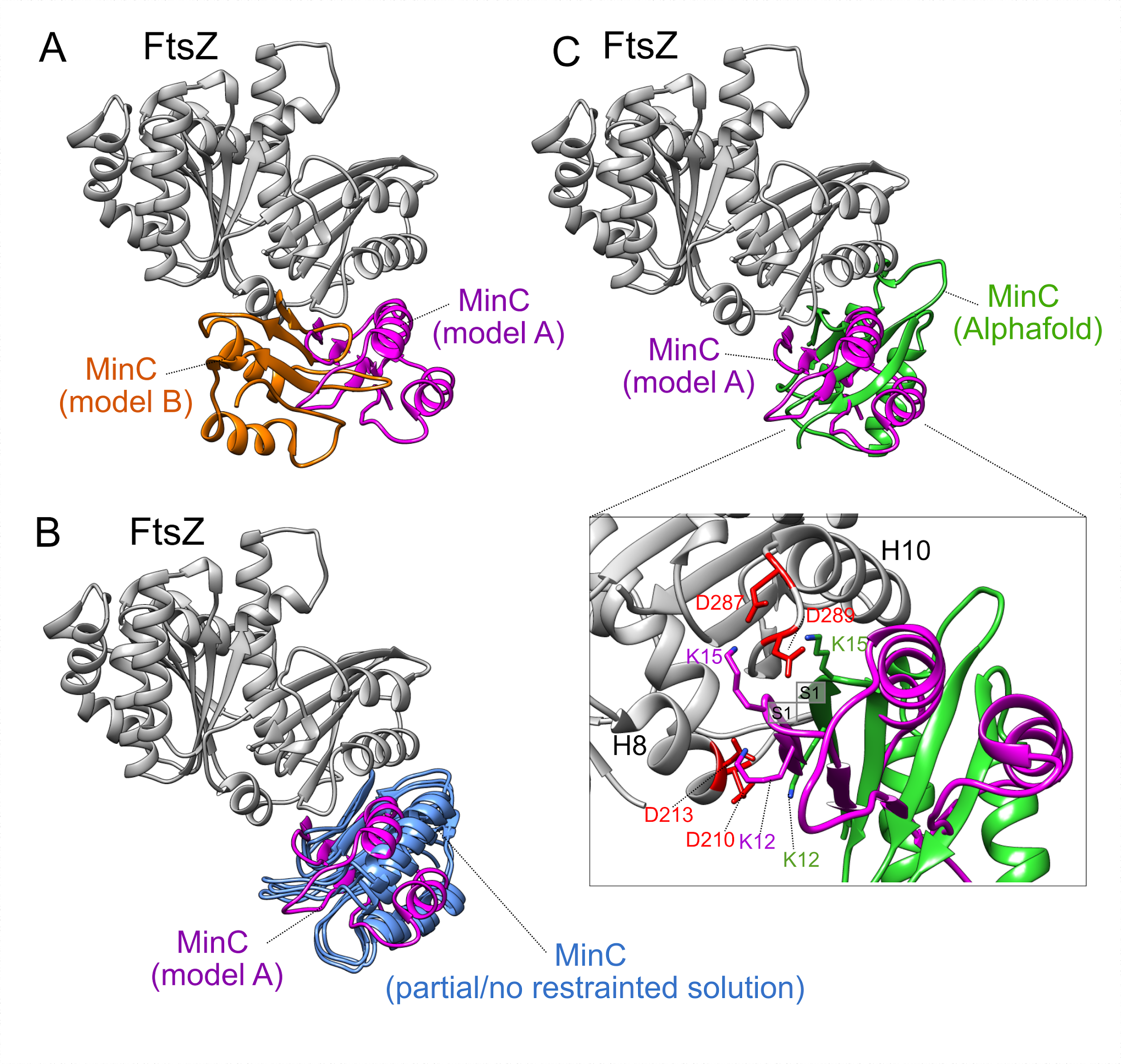


***Supplemental Figure 4****. Comparison among 3D structural models of FtsZ:MinC complex. (A) Structural comparison between FtsZ:MinC 3D models (model A, in magenta, and model B, in orange) modeled by protein-protein docking with ClusPro server, using constraints based on experimental data. Models are aligned by FtsZ structure. (B) Structural comparison between model A (in magenta), generated in ClusPro server with constraints, and three other best ranked models (in blue), generated in ClusPro server with no constraints (Model C), with only MinC constraints (Model D), or with only FtsZ constraints (Model E). Since all these alternative models C, D and E, adopts a similar interaction position, we did not name them in the structure, for clarity. (C) Structural comparison between model A (in magenta), generated in ClusPro server with constraints, and the structural model generated by AlphaFold-Multimer (in green). In inset, a zoomed view of contacts between positively charged residues of MinC S1 and negatively charged residues of FtsZ. Important residues in this contact adopt similar positions in both proposed models.*


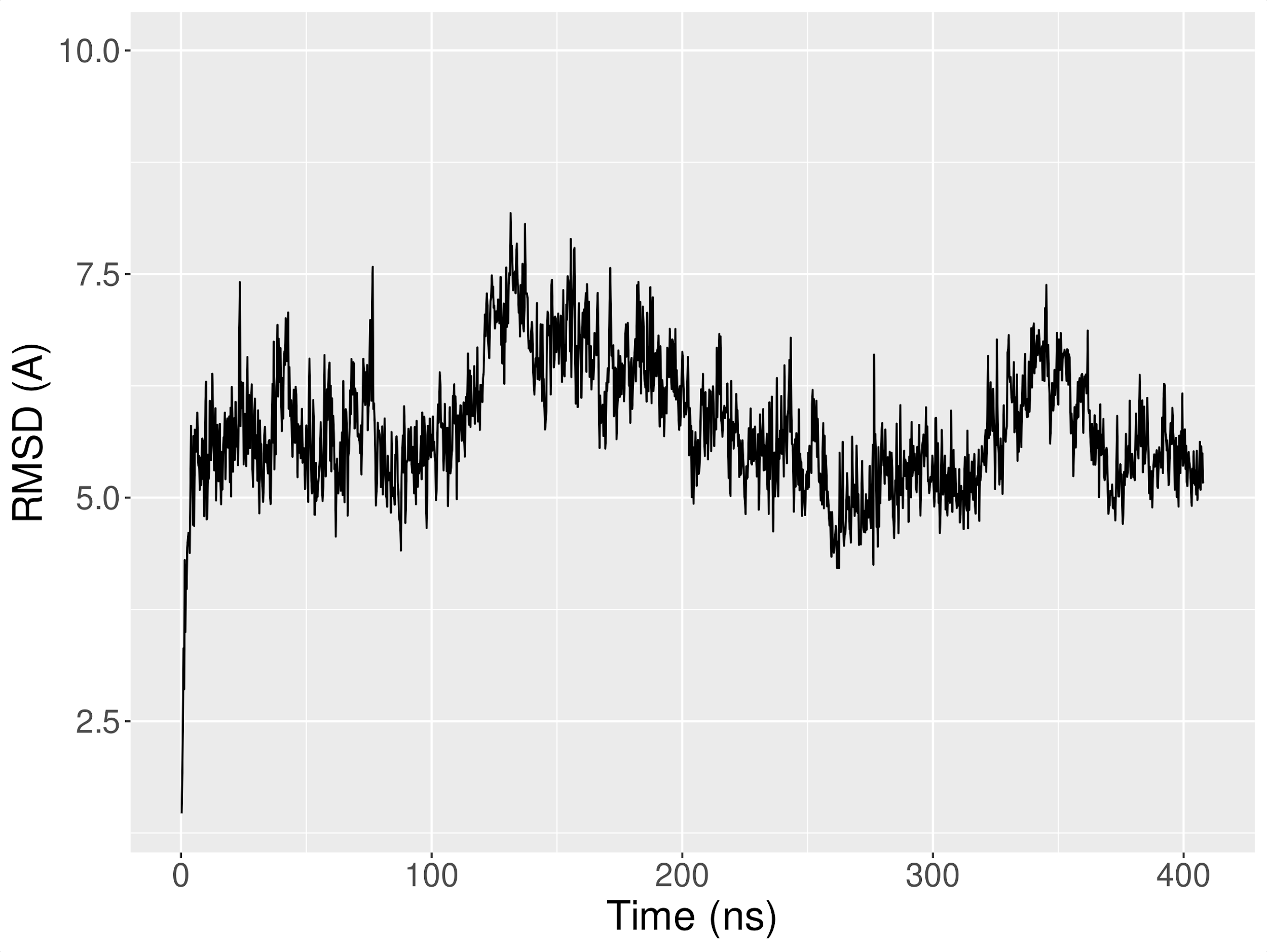


***Supplemental Figure 5****. Root-mean-squared deviation (RMSD) of MinC during the 400 ns molecular dynamics simulation.* *The backbone MinC RMSD was calculated in VMD software and the system was aligned by FtsZ proteins using the first frame as reference.*


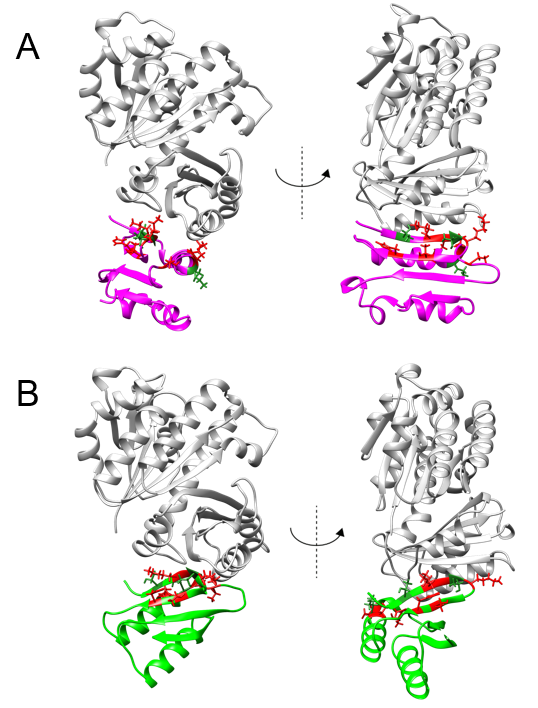


***Supplemental Figure 6.*** *Mapping of E. coli MinC^N^ mutations of Table S5 onto the structure of the NMR-docking complex (A) and Alpha Fold Multimer complex (B). Mutations are highlighted as sticks, with those that disrupt MinC^N^ function colored red and those without effect colored dark green.* **Supplemental References**

Blasios, V., Bisson-Filho, A.W., Castellen, P., Nogueira, M.L., Bettini, J., Portugal, R.V., Zeri, A.C., Gueiros-Filho, F.J., 2013. Genetic and biochemical characterization of the MinC-FtsZ interaction in Bacillus subtilis. PLoS One 8, e60690.

Castellen, P., Sforça, M.L., Gueiros-Filho, F.J., De Mattos Zeri, A.C., 2015. Backbone and side chain NMR assignments for the N-terminal domain of the cell division regulator MinC from Bacillus subtilis. Biomol. NMR Assign. 9, 1–5. https://doi.org/10.1007/s12104-013-9534-y

Park, K.-T., Dajkovic, A., Wissel, M., Du, S., Lutkenhaus, J., 2018. MinC and FtsZ mutant analysis provides insight into MinC/MinD-mediated Z ring disassembly. J. Biol. Chem. 293, 5834–5846. https://doi.org/10.1074/jbc.M117.815894

Youngman, P.J., Perkins, J.B., Losick, R., 1983. Genetic transposition and insertional mutagenesis in Bacillus subtilis with Streptococcus faecalis transposon Tn917. Proc Natl Acad Sci U A 80, 2305–9.
